# Influence of imaging method on fat fraction estimation for assessing bone marrow in metastatic prostate cancer

**DOI:** 10.1007/s00330-025-11564-7

**Published:** 2025-04-11

**Authors:** Yassine N. Azma, Nada Boci, Katarzyna Abramowicz, Luca Russo, Matthew R. Orton, Nina Tunariu, Dow-Mu Koh, Geoffrey Charles-Edwards, David J. Collins, Jessica M. Winfield

**Affiliations:** 1 MRI Unit, The Royal Marsden NHS Foundation Trust, London, UK; 2 Division of Radiotherapy and Imaging, The Institute of Cancer Research, London, UK; 3 Dipartimento Diagnostica per Immagini, Radioterapia Oncologica ed Ematologia, Fondazione Policlinico Universitario A. Gemelli IRCCS, Rome, Italy

**Keywords:** Bone marrow, Prostatic neoplasms, Magnetic resonance imaging

## Abstract

**Objective:**

This study aimed to assess the accuracy of fat fraction estimation with clinically available Dixon sequences in normal-appearing marrow and bone metastases in the pelvis of metastatic prostate cancer patients.

**Methods:**

A prospective single-centre study was conducted with metastatic prostate cancer patients and healthy volunteers. Linearity and bias of fat fraction estimates from clinically available Dixon sequences were assessed against a 6-point PDw gradient echo (q-Dixon) sequence measuring the reference standard proton density fat fraction. Lesion fat fraction estimates were cross-compared using the Friedman test. Repeatability in volunteers was evaluated with Bland-Altman plots. Sensitivity of fat fraction estimates using TSE-Dixon sequences to specific absorption rate (SAR) related modifications were evaluated with correlation plots.

**Results:**

Thirty-three patients were recruited for this study. Significant (*p* < 0.05) absolute bias (12.4%) was demonstrated in the T1-weighted (T1w) Dixon measurements against the q-Dixon. Significant differences (*p* < 0.05) between fat fraction estimates provided by the T1w Dixon and PDw Dixon sequences were observed in 13 active and 6 treated lesions. Repeatability coefficients for fat fraction estimates ranged from 5.9 to 9.0% in the pelvic tissues of healthy volunteers. Reduction of slice number with repetition time for SAR had the greatest effect, reaching a maximum difference in fat fraction of 14.7% from the q-Dixon for the T2w-TSE Dixon in bone marrow.

**Conclusions:**

T1w Dixon methods can detect post-treatment changes but remain confounded by relaxation time biases. While all Dixon methods showed good repeatability, careful choice of SAR-related modifications is critical to maintaining accuracy for PD- and T2-weighted TSE sequences.

**Key Points:**

***Question*** *The clinical validity of signal-weighted fat fraction estimates versus proton density fat fraction for characterising metastatic bone lesions has not been fully assessed.*

***Findings*** *T1-weighted Dixon sequences in line with whole-body MRI international guidelines demonstrate significant fat fraction bias, particularly in lesions and muscle.*

***Clinical relevance*** *Fat fraction estimation using T1-weighted Dixon sequences recommended in international guidelines are highly sensitive to relaxation time biases, making underlying physiological changes potentially ambiguous.*

**Graphical Abstract:**

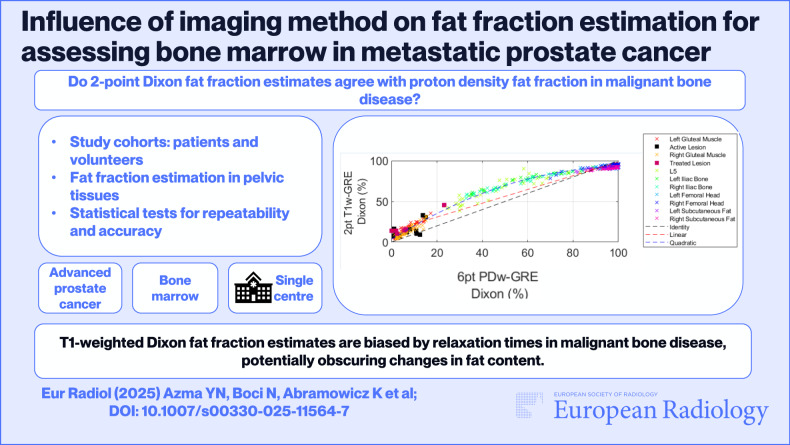

## Introduction

In whole-body MRI (WB-MRI), Dixon MRI and diffusion-weighted MRI (DWI) are integral techniques for the assessment of disease extent and treatment response of bone disease in advanced prostate cancer [1] and multiple myeloma [2]. Dixon MRI enables fat-water separation, allowing qualitative and quantitative assessment of the displacement of marrow fat by tumour growth. Increase in the estimated fat fraction of bone metastases has been shown to reflect response in patients with metastatic prostate cancer [3] and has been demonstrated to precede changes of apparent diffusion coefficient (ADC) in patients with multiple myeloma who responded to treatment [4]. These two techniques have been demonstrated to provide information that correlates well with histological features of bone metastases [3], enabling virtual biopsy of bone metastases and targeted selection of appropriate lesions for CT-guided biopsy when needed [5].

WB-MRI is a long protocol. It typically requires at least 45 minutes in total, with a large portion of its duration dedicated to acquiring DWI of sufficient signal-to-noise ratio and resolution. Any further additions must be carefully considered to maximise patient benefit given the established clinical value of DWI and Dixon imaging [6]. Clinical guidelines for WB-MRI, such as Metastasis Reporting and Data System for Prostate Cancer (MET-RADS-P) and Myeloma Response Assessment and Diagnosis System (MY-RADS) [1, 2], recommend a 2-point gradient echo T1-weighted (T1w) Dixon sequence. This sequence achieves both fat-water separation and fat fraction estimation, providing T1w anatomical imaging alongside relative fat quantification without additional acquisition time. However, this fat fraction estimate is known to be subject to multiple sources of bias, including T1 relaxation [7, 8], T2* decay [9, 10], noise bias [8, 11], field inhomogeneity [12], the spectral complexity of fat [10, 13], and gradient readout scheme [14].

Sequences and associated reconstructions that correct for these biases produce the proton density fat fraction (PDFF) and are available in commercial packages from every major MR vendor. The PDFF is well-established as a reference standard for fat estimation in liver [15]. There is a paucity of literature that has investigated the biases between implementations of PDFF and long-standing 2-point Dixon methods in bone marrow imaging. Bainbridge et al have conducted a multi-centre study, demonstrating that biases seen within healthy marrow data acquired with a 2-point Dixon can be minimised with a simple offline correction [16]. The generalisability of these results to malignant bone disease remains unseen.

Consequently, this study aimed to assess whether there are significant differences in fat fraction estimated from clinically available Dixon sequences and a sequence that produced the PDFF, a quantitative imaging biomarker for tissue fat concentration and reference standard for this study [17], both in healthy tissues and malignant bone lesions. This study also evaluated the repeatability of fat fraction estimates of each imaging method to inform longitudinal studies of treatment response in bone marrow disease.

## Materials and methods

### Study populations

This prospective single-centre study was approved by a national research ethics committee (NCT05118555, clinicaltrials.gov). All patients provided verbal consent (written consent was waived by the institution) for the acquisition of additional research imaging data as part of routine clinical examinations, and all healthy volunteers provided written consent to participate in the study. This study consisted of three cohorts, which will be referred to as the patient cohort, volunteer cohort 1, and volunteer cohort 2.

A power calculation (one-sided *t*-test, β = 0.1, α = 0.05) was performed to determine the minimum number of subjects required to detect a significant difference in muscle fat fraction measured by T1w and proton-density weighted (PDw) Dixon sequences in an initial volunteer study. Muscle was chosen because it has a comparable fat fraction to active metastatic disease in bone marrow [3]. The minimum number of patients required for the study was calculated to be 28. Further details regarding the initial phantom and initial volunteer study can be found in Supplementary Figs. 1 and 2.

The patient cohort consisted of patients with metastatic prostate cancer who were scheduled for whole-body MRI examinations on one scanner and were invited to participate in the study. Patients with metal implants in the pelvis were excluded from this study. No biopsy data were available or specifically acquired for this cohort.

Volunteer cohort 1 consisted of healthy volunteers who were recruited to establish the repeatability of a variety of clinically available sequences for fat fraction estimation.

Volunteer cohort 2 was recruited to further investigate the performance of fat quantification in turbo-spin echo (TSE) Dixon sequences with protocol modifications that may be implemented clinically by radiographers/technicians to deal with specific absorption rate (SAR) or time constraints. Volunteers who had participated in cohort 1 were allowed to volunteer again in cohort 2.

### Image acquisition

All subjects were scanned on a 1.5-T scanner (MAGNETOM Sola, Siemens Healthcare). Scan volumes for the patient and first volunteer cohort were a single station that covered from L5 to the femoral heads. For the second volunteer cohort, the scan volume covered from the iliac bones to the upper femoral shafts. All volumes shared matched slice positions with the DWI sequence for the patient cohort.

The patient cohort received their routine clinical WB examination before the additional research sequences which were the same as acquired in volunteer cohort 1. These include a 2-point T1w gradient echo Dixon in line with international guidelines, which is known to exhibit biases to T1, T2*, and incomplete modelling of the fat signal, and is currently used at our institution. A 2-point PDw gradient echo Dixon, which aimed to minimise the T1 bias by lowering the flip angle. A 6-point PDw gradient echo Dixon (from hereon q-Dixon) was acquired using the scanner manufacturer’s LiverLab package, which uses a hybrid fitting approach with a seven-peak spectral model for fat and T2* correction [18]. The q-Dixon, which has the most sources of biases minimised, was used to provide PDFF estimates using an established clinical imaging sequence. 3-point TSE Dixon sequences were also included, owing to their emerging use in bone metastasis imaging [19–21]. They differed in echo time with varying degrees of T2-weighting, resulting in differing sensitivities to T2-related bias. The sequence parameters for volunteer cohort 1 are detailed in Table 1.

**Table 1:** Sequence parameters for the second volunteer cohort

MRI acquisition parameters—protocol for patient and volunteer 1 cohorts							
Group	Sequence	TE_1_/ΔTE/N_TE_ (ms)	TR (ms)	Flip angle (°)	Number of slices	Acquisition time (min:ss)	Other
3D GRE Dixon	2pt T1w	2.39/2.38/2	7.1	20	44	00:19	CAIPIRINHA 2 × 2 (85% PE)
	2pt PDw			3			
	6pt PDw (q-Dixon)	1.20/1.27/6	9.0	4		00:15	CAIPIRINHA 1 × 3 (85% PE, 50% SE)
2D TSE Dixon	3pt T2w	89 (−π/0/π)	7980	90/150	36	02:17	GRAPPA 2 (85% PE), ETL = 16
	3pt T2w (volunteer only)	25 (−π/0/π)					
	3pt PDw	13 (−π/0/π)					
2D DWI	3-scan trace EPSE-DWI (patient only)	71	5880	90	40 (per station)	03:51 (per station)	b = 50, 600, 900 (s/mm^2^) | averages = 3, 3, 6 STIR, Twice-refocused spin echo (bipolar)

All sequences were acquired in the axial plane

For TSE sequences, the numbers in brackets for the echo time scheme represent the phase shifts created between fat and water

*TE*_*1*_ first echo time, *ΔTE* inter-echo spacing, *N*_*TE*_ number of echoes, *PE* phase encoding sampling, *SE* slice encoding sampling (3D only), *EPSE-DWI* echo-planar spin echo diffusion-weighted imaging, *GRE* gradient echo, *TSE* turbo-spin echo, *ETL* echo train length, *CAIPIRINHA* controlled aliasing in parallel imaging results in higher acceleration, *GRAPPA* generalised autocalibrating partial parallel acquisition, *STIR* short T1 inversion recovery, *TR* Repetition time

The protocol for volunteer cohort 2 included variations in the number of slices, echo train length, and refocusing flip angle for both T2- and PD-weighted TSE sequences. The sequence parameters used for volunteer cohort 2 are further detailed in Table 2.

**Table 2:** Sequence parameters for the second volunteer cohort

MRI acquisition parameters—protocol for volunteer cohort 2							
Sequence	TE_1_ (ms)	TR (ms)	Refocusing flip angle (°)	Number of slices	Acquisition time (min:ss)	Echo train length	Other
2D 3pt T2w-TSE Dixon	89 (−π/0/π)	7980	150	36	02:17	16	GRAPPA 2 (PE 85%)
		4310			02:24	8	
		15,320			02:19	32	
		3990		18	01:09	16	
		2000		9	00:36	16	
2D 3pt PDw-TSE Dixon	13 (−π/0/π)	7980		36	02:17	16	
		4310			02:24	8	
		15,320			02:19	32	
		3990		18	01:09	16	
		2000		9	00:36		
2D 3pt T2w-TSE Dixon	89 (−π/0/π)	7980	180	36	02:17		
			160				
			150				
			140				

All sequences were acquired in the axial plane

For TSE sequences, the numbers in brackets for the echo time scheme represent the phase shifts created between fat and water

*TE*_*1*_ first echo time, *ΔTE* inter-echo spacing, *N*_*TE*_ number of echoes, *PE* phase encoding undersampling, *TSE* turbo-spin echo, ETL echo train length, *GRAPPA* generalised autocalibrating partial parallel acquisition, *TR* Repetition time

### Imaging analysis

Regions of interest (ROIs) were outlined using Horos v4.0.0 RC5 (horosproject.org) in the following healthy tissues in patients and healthy volunteers of cohort 1: left and right gluteal muscle, left and right posterior iliac bones, left and right femoral heads, left and right subcutaneous fat, and the L5 vertebrae (area > 1 cm^2^). These ROIs were drawn on the 2-point T1w-Dixon fat fraction maps by an MRI physicist (Y.A., with 3 years of experience in clinical imaging). For volunteer cohort 2, only the right and left acetabulum, femoral heads, gluteal muscle, and subcutaneous fat were outlined.

Using the fat fraction maps in conjunction with ADC maps calculated by the manufacturer from DWI (b = 50, 600, 900 s/mm^2^), bone lesions were outlined separately on both the fat fraction and ADC maps owing to the varying geometric distortions between the image series. Image registration was not employed due to disparate signal properties between DWI and Dixon imaging alongside heterogeneity of the imaged skeleton in the scanned population. ROIs were drawn by an MRI physicist (Y.A.) and validated by two radiologists with > 10 years (N.T.) and 2 years (K.A.) of experience in whole-body imaging for advanced prostate cancer.

Only one active and one treated lesion were taken from each patient to avoid multiplicity effects. Each lesion was between the femoral heads and L5, free of significant motion artefacts in the Dixon sequences, and of at least 1 cm^2^ area in a single slice at the time of imaging. If a patient had a CT acquired within three months of their WB-MRI, this CT was then used to further classify lesions as either sclerotic or lytic. Details of this classification can be found in Table 3.

**Table 3:** Lesion classifications

Lesion types	
Classification	#
Patients with suitable active (and/or) treated lesion	18 of 33
Total lesions	19
Active/treated	13/6
Lesions with CT within 3 months	10 of 19
Lytic/sclerotic	1/9

For lesions, the 2-point gradient echo PDw and T1w Dixon sequences were used to calculate T1 maps using the DESPOT1 approach [22]. No B1 correction was applied. ADC maps were calculated using a mono-exponential fit using all three b-values (Levenberg-Marquardt algorithm in lsqcurvefit, MATLAB version 2022b, The Mathworks). Lesions were determined to be active if they had an ADC of less than 800 × 10^−^^6^ mm^2^/s and abnormal fat fraction or treated if they had an ADC of greater than 1400 × 10^−^^6^ mm^2^/s and abnormal fat fraction. Fat fraction values of 20% or lower from the 2-point T1w Dixon sequence were classified as abnormal fat fractions [5]. Finally, R2* (the reciprocal of T2*) was acquired as an additional output of the manufacturer’s PDFF sequence. All fat-water separation was performed inline by the scanner’s reconstruction software. Fat fraction was calculated for all sequences offline in accordance with the magnitude discrimination method of Liu et al [8].

**Fig. 1:**
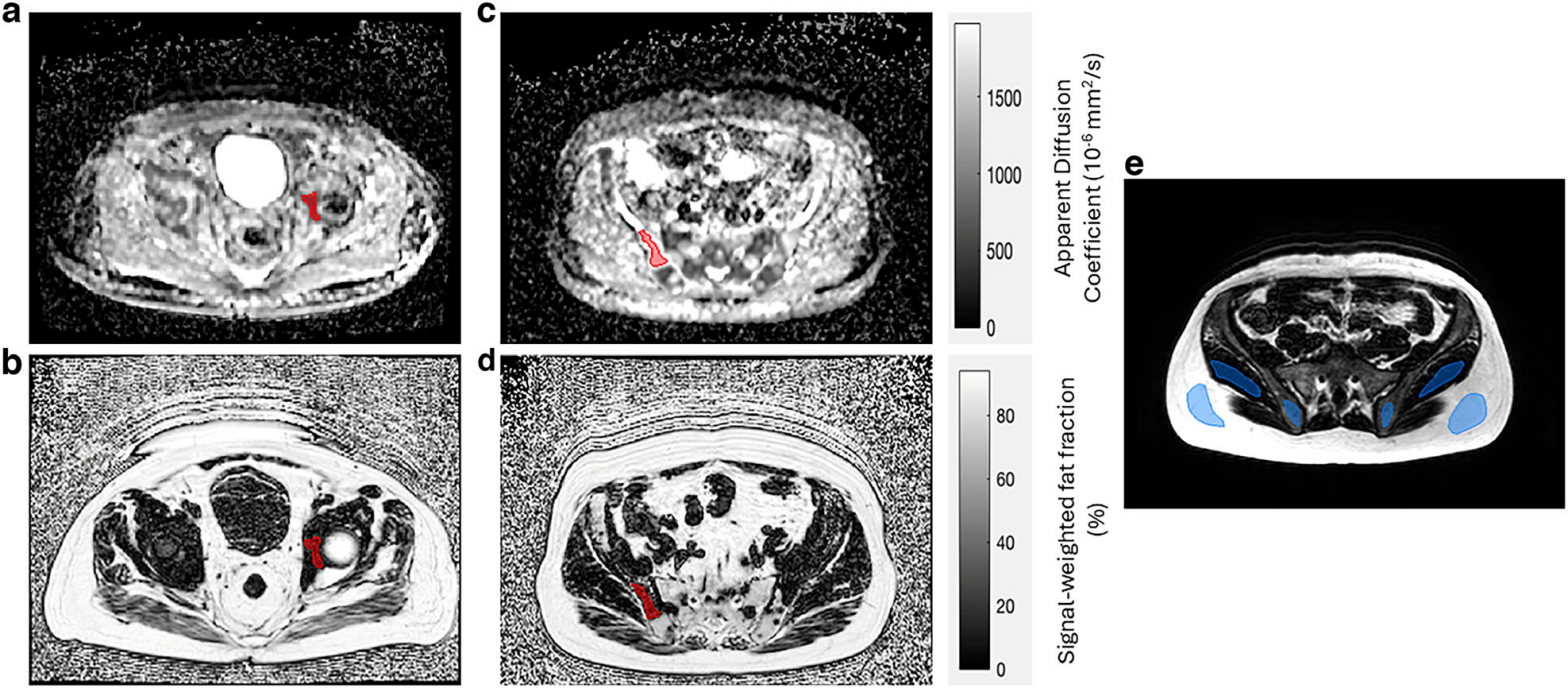
**a** Apparent diffusion coefficient (ADC) map of a central slice through an active left acetabular bone lesion (ADC median = 694.7 × 10^−^^6^ mm^2^/s). **b** T1w fat fraction (FF) map of the slice containing the same active left acetabular bone lesion (T1w-FF = 14.2%). **c** ADC map of a central slice through a treated right posterior iliac bone lesion (ADC median = 1750.1 × 10^−^^6^ mm^2^/s). **d** T1w-FF map of the slice containing the same treated right iliac bone lesion (T1w-FF = 15.6%). **e** ROI positions in subcutaneous fat, posterior iliac bones, and gluteal muscle

All ROIs were converted to Digital Imaging and Communications in Medicine–Radiotherapy (DICOM-RT) using pyOsirix [23] and imported into MATLAB as binary masks. ROIs outlined on the fat fraction maps were copied across to the other fat fraction maps produced by other Dixon sequences in MATLAB. Median values for all quantitative outputs were reported from each ROI. Examples of images and associated ROI placements for active and treated disease in the patient cohort and the volunteer cohort 1 are shown in Fig. 1.

**Fig. 2:**
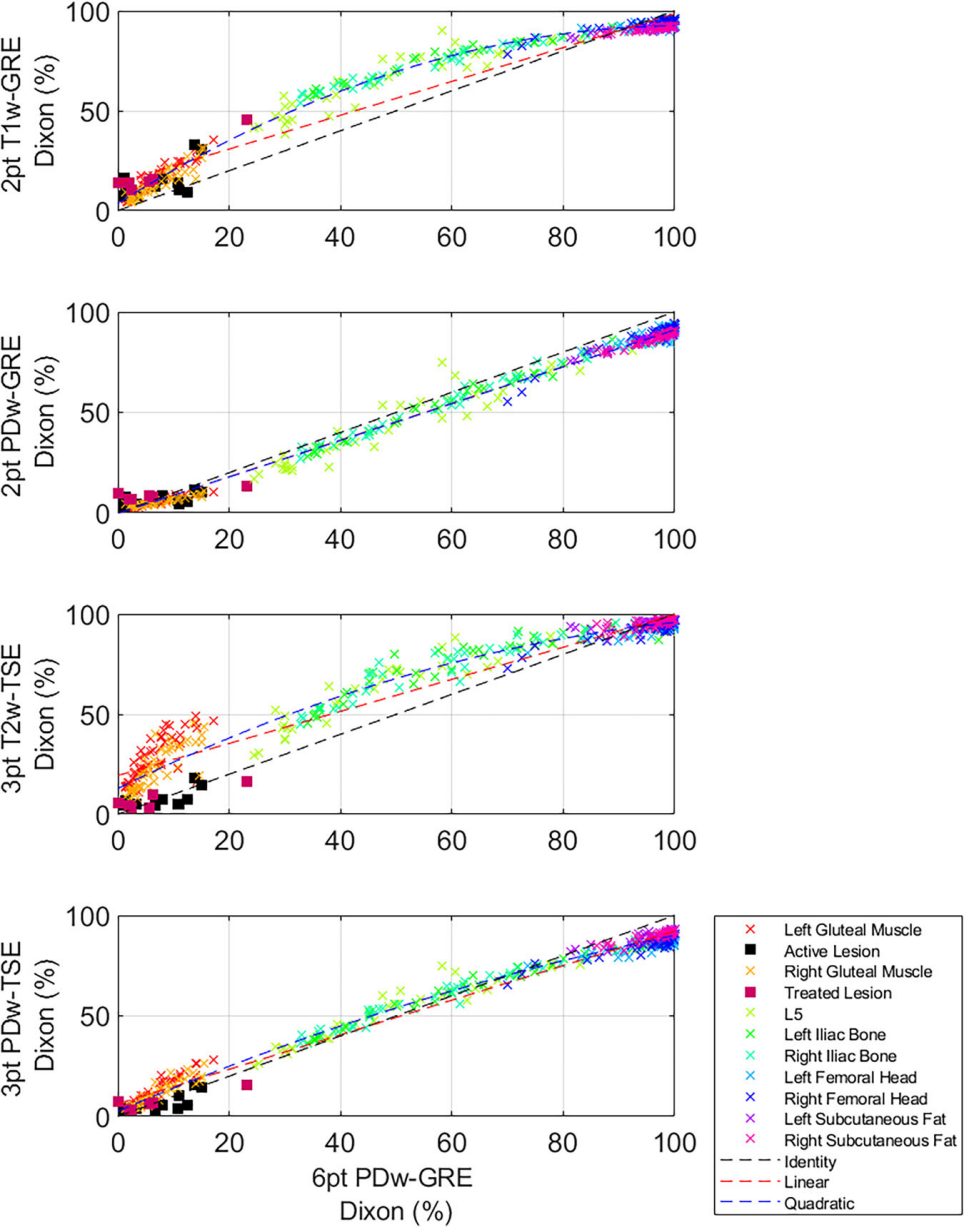
Correlation plots of each Dixon sequence against the PDFF reference, q-Dixon. The volunteer and patient data have been pooled for the characterisation of the entire fat fraction range. Red dashed lines indicate linear fits, blue dashed lines indicate quadratic fits, and the black dashed line indicates the line of identity

### Statistical methods

Linearity and bias were evaluated for the combined patient and volunteer cohort 1 datasets for each method compared to the proton-density weighted Dixon sequences, including the PDFF as the reference standard, over the entirety of the fat fraction range.

Regression analysis was used to compare the accuracy of each T1w and T2-weighted (T2w) Dixon sequence against PD-weighted Dixon sequences. Linearity and bias were assessed via linear least squares regression with a linear fit to obtain the slope and intercept. Curvature was assessed with a quadratic polynomial fit via the size of the x^2^ coefficient to compare different degrees of nonlinear bias.

Accuracy of lesion fat fraction estimates was subject to cross-comparison to determine the existence of any significant differences using the Friedman test for repeated measures.

Two-sample *t*-tests were used to assess whether there was a difference in median T1_water_ or median R2* values between active and treated lesions. The correlation between CT-derived Hounsfield units and R2* was also assessed.

For the volunteer cohort 1, Bland-Altman analysis [24] was used to determine the limits of agreement and repeatability coefficient for each sequence. Fat fraction estimates from different T2w- and PDw-TSE Dixon sequences (volunteer cohort 2) were compared using correlation plots to assess agreement.

## Results

### Volunteer and patient characteristics

The patient cohort was composed of thirty-three male patients (median age 71 years, range 57–87 years) with clinically diagnosed advanced prostate cancer recruited from April 2022 to May 2023. There was no distinction made regarding the status of their disease at the time of their examination. Ultimately, 13 active lesions and 6 treated lesions met the requirements described in the “Materials and methods” section and were included in the analysis. Patients without suitable lesions still contributed normal-appearing tissues.

Volunteer cohort 1 comprised ten healthy volunteers (5 female, 5 male; median age 35 years, range 25 to 45 years). Volunteer cohort 2 comprised five healthy volunteers (2 female, 3 male; median age 28 years, range 27 to 41 years).

PDw methods systematically underestimate fat fraction compared to the PDFF reference standard provided by the q-Dixon. 3pt T2w-TSE Dixon and 2pt T1w-gradient echo Dixon sequences show marked nonlinear overestimation of fat fraction in muscle and bone marrow, though they also underestimate fat fraction for subcutaneous fat. Despite good agreement in lesional fat fraction, the T2-TSE Dixon exhibits striking overestimation in the muscle (fat fraction = 30.0%) compared with the q-Dixon (7.3%) (Fig. 2). Plots showing all comparisons of all combinations of fat fraction estimates are shown in Supplementary Figs. 3–5.

With regression analysis, good agreement can be seen against the q-Dixon for the 2-point PD-weighted Dixon (slope = 1.12, intercept = 0.80%, curvature = −2e-4). This is in contrast to the clinical standard of T1-weighted Dixon imaging (slope = 1.12, intercept = −12.41%, curvature = 1.28e-2) (Table 4).

**Table 4:** Intercept, bias, and curvature results of linear and quadratic fits, respectively, of various Dixon sequences against the PDFF reference, as seen in Fig. 2

Fat fraction regression parameters	
Intercept	q-Dixon
2pt T1w-GRE Dixon	−12.41 [−14.26, −10.55]***
2pt PDw-GRE Dixon	0.80 [0.23, 1.36]**
3pt T2w-TSE Dixon	−19.30 [−21.40, −17.21]***
3pt PDw-TSE Dixon	−6.30 [−7.18, −5.41]***
**Slope**	
2pt T1w-GRE Dixon	1.12 [1.09, 1.14]***
2pt PDw-GRE Dixon	1.09 [1.08, 1.10]***
3pt T2w-TSE Dixon	1.17 [1.15, 1.20]***
3pt PDw-TSE Dixon	1.15 [1.13, 1.16]***
**Curvature (× 100)**	
2pt T1w-GRE Dixon	1.28 [1.20, 1.36]***
2pt PDw-GRE Dixon	−0.02 [−0.07, 0.03] ns
3pt T2w-TSE Dixon	1.09 [1.00, 1.18]***
3pt PDw-TSE Dixon	0.43 [0.37, 0.48]***

All x^2^ coefficients in the curvature section of the table have been multiplied by 100 to enhance readability. 95% confidence intervals are reported for each parameter in square brackets

** *p* < 0.01, *** *p* < 0.001, ns not significant

**Fig. 3:**
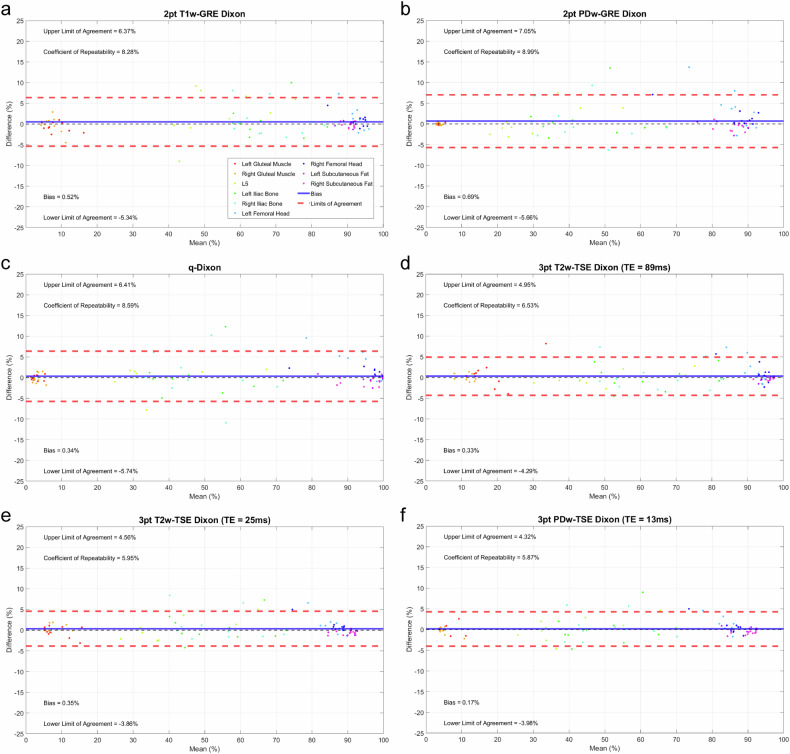
Bland-Altman plots for each Dixon sequence ran in healthy volunteers (volunteer cohort 1). . **a** 2pt T1w-Dixon. **b** 2pt PDw-Dixon. **c** q-Dixon. **d** 3pt T2w-TSE Dixon (TE = 89 ms). **e** 3pt T2w-TSE Dixon (TE = 25 ms). **f** 3pt PDw-TSE Dixon (TE = 13 ms).  Each individual plot consists of the paired measurements for different anatomical sites of all volunteers acquired during a scan-rescan assessment of repeatability. The bias is reported as the mean difference between paired measurements. The solid blue line indicates the bias, as previously mentioned. The dashed red lines show the upper and lower limits of agreement

Repeatability coefficients for each fat fraction method across all tissue types varied between 5.9 and 9.0% (Fig. 3).

**Fig. 4:**
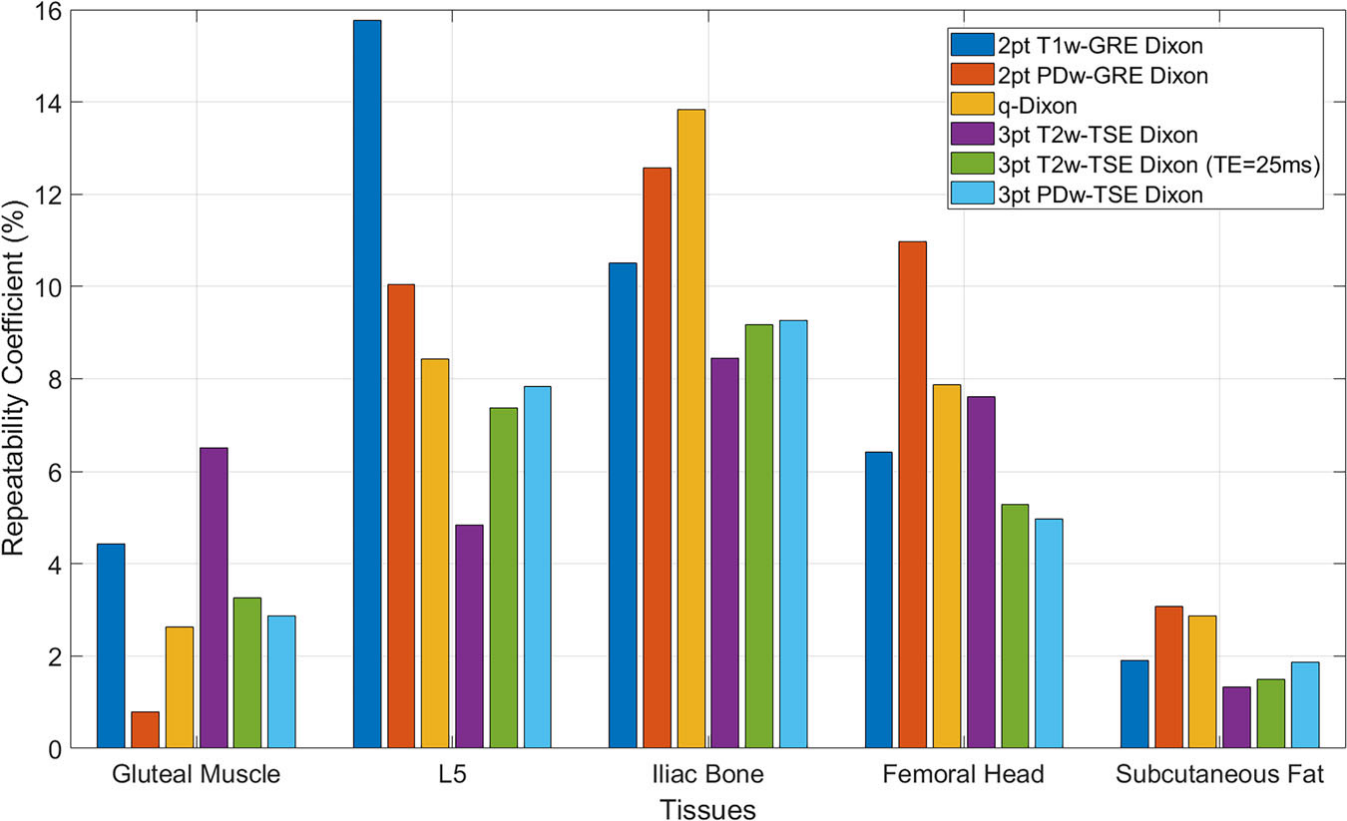
Repeatability coefficient for each tissue type by Dixon sequence

Repeatability of all Dixon sequences is highest for tissues that are either purely water or fat signal. When tissues have fat and water in near-equal proportion, repeatability falls (Fig. 4).

**Fig. 5:**
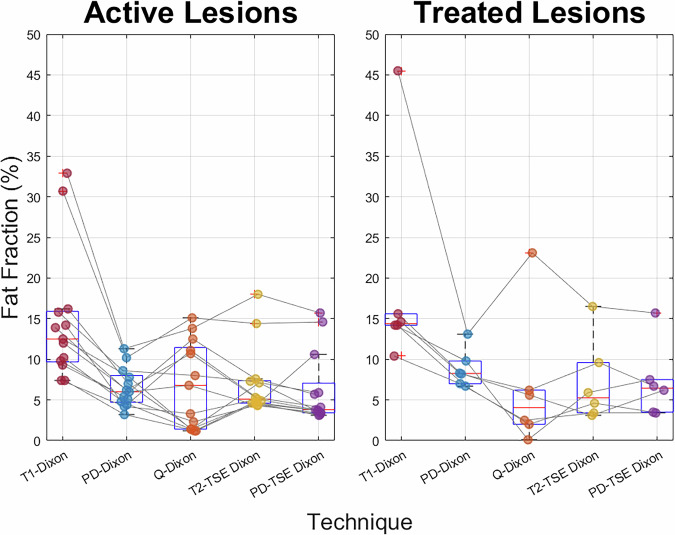
Boxplots of median fat fraction values in a) active (13 patients) and b) treated lesions (6 patients) using each of the Dixon sequences in the patient protocol. Lines from data point to data point show a specific lesion’s variation with Dixon sequence

There were significant differences (Friedman test *p* < 0.05) between the fat fraction of active lesions measured with the 2-point T1w-gradient echo Dixon sequence and all other methods in the patient cohort. For treated lesions, there is a significant difference (*p* < 0.05) between only the 2-point T1w-gradient echo Dixon and the q-Dixon and 3-point PDw-TSE Dixon sequences (Fig. 5).

There was no significant difference in either T1_water_ or R2* between active and treated lesions (*p* > 0.05) (Supplementary Fig. 4). A strong positive correlation (*r* = 0.78, *p* < 0.01) is seen between the R2* of bone marrow lesions and Hounsfield unit (Supplementary Fig. 5).

**Fig. 6:**
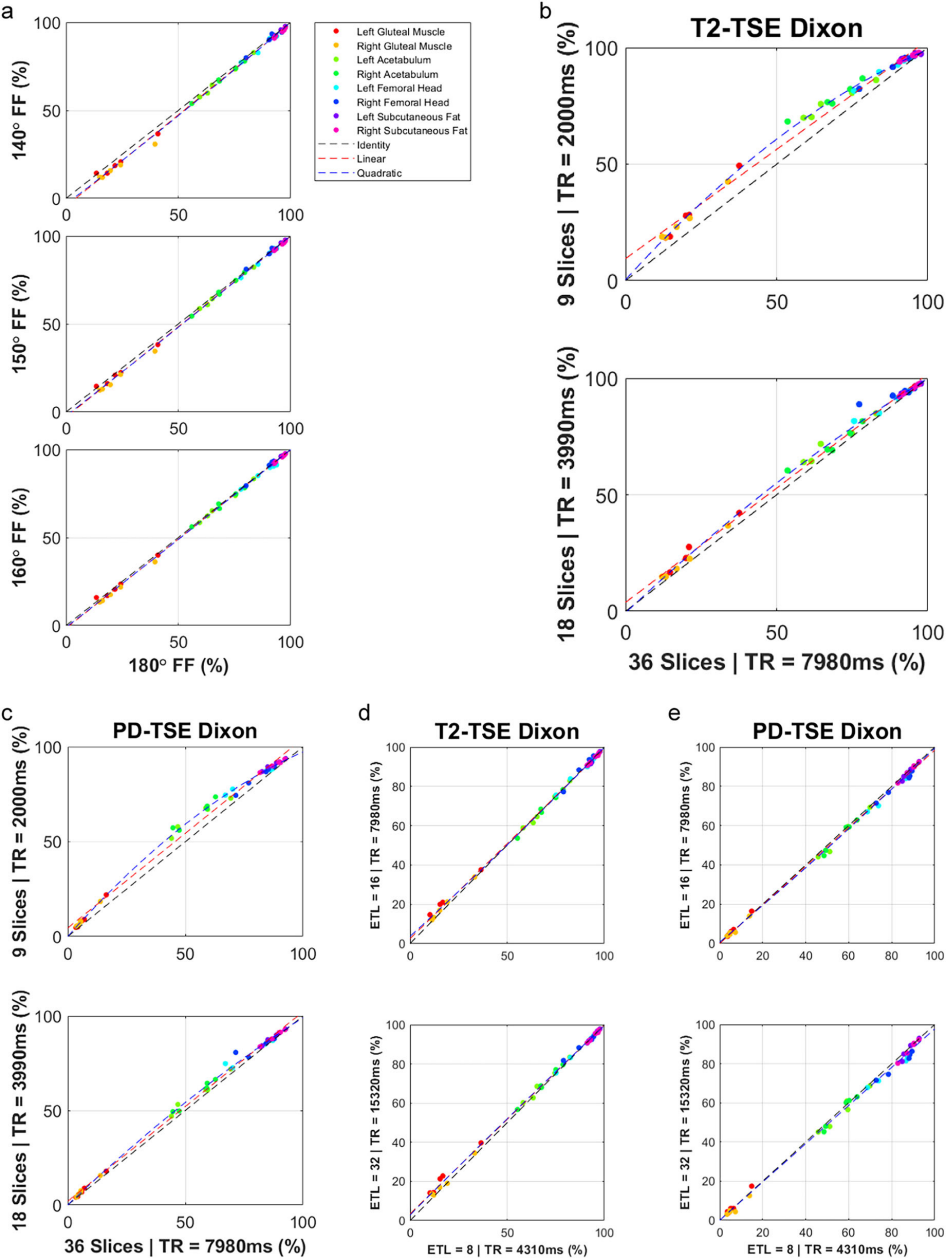
Correlation plots demonstrating agreement of TSE Dixon sequences used for fat quantification in healthy volunteers (volunteer cohort 2). **a** The effects of varying refocusing angle for a T2-TSE Dixon, **b**, **c** the effects of varying slice number for T2- and PD-weighted TSE Dixon sequences. **d**, **e** The effects of varying echo train length (ETL) for T2- and PD-weighted TSE Dixon sequences

Reducing the refocusing angle of a T2-TSE Dixon results in increasing disagreement in FF estimates compared with the 180-degree ideal predominantly in the muscle (Fig. 6a). Reducing the number of slices, in conjunction with a reduction in repetition time, leads to increasing nonlinear T1 bias for both T2- and PD-weighted TSE sequences respectively (Fig. 6b, c). Modifying the echo train length from 16, in conjunction with repetition time, leads to marginal effects (Fig. 6d, e).

## Discussion

The purpose of this study was to provide a comparative analysis of the performance of various clinically available Dixon sequences to a PDFF reference. This study has demonstrated that there are significant differences in fat fraction estimation, in both healthy tissues and lesions, with all sequences and the PDFF reference in active lesions. Repeatability was shown to be good for all approaches, though it was worst for the gradient-echo approaches and for tissues where fat and water are in near-equal proportion. TSE sequences are largely insensitive to reduction of the refocusing flip angle but show T1-related biases when the repetition time is lowered in conjunction with changes to echo train length and slice number.

Our findings align with prior studies highlighting the limitations of T1-weighted gradient echo Dixon sequences in accurately estimating fat fraction. The limits of agreement across the entire fat fraction range reported in this study were comparable for the TSE sequences and slightly worse for the spoiled gradient echo sequences to those reported in liver studies [25]. However, when considering individual tissues, bone marrow repeatability is worse. Bone marrow is a notably challenging region for fat quantification due to the trabecular environment causing highly accelerated susceptibility-induced dephasing [26]. Fat-water separation is most difficult when water and fat are in near-equal proportion [27–29], which could explain the degraded repeatability of all methods employed in the hematopoietic bone marrow in L5, particularly for the q-Dixon’s hybrid fitting approach including a magnitude-based component [18]. TSE Dixon methods are more repeatable, potentially due to the additional echo and fewer unknowns compared with the 2pt and 6pt gradient echo methods. They have been explored in several studies [19, 20] for the detection of skeletal metastasis and myeloma, though they have been demonstrated in this study to exhibit T1 and T2-related biases. Muscle is particularly sensitive to noise-related effects, owing to its short T2 [30, 31]. Such biases should be considered when developing methods for fat quantification in musculoskeletal applications that interrogate fatty infiltration of muscle, such as Duchenne’s muscular dystrophy, where fat content in the muscle is prognostic for loss of ambulation [32].

The clinical implications of this study for the optimisation of imaging protocols in oncology applications largely concern the high degree of T1 bias of the 2pt T1w-gradient echo Dixon. The estimated fat fraction produced by T1-weighted Dixon sequences in whole-body oncology imaging is increasing in importance for monitoring disease and assessing treatment response [3, 4, 33, 34]. Treatment may induce T1 relaxation time changes [35, 36] which would lead to a change in presentation of the bias observed by a T1-weighted Dixon. It is plausible that there may be instances where the measured fat fraction produced by a T1-weighted Dixon sequence may change post-treatment, suggesting a change in tissue fat concentration, but only the T1 of water has changed. For this reason, PD-weighted Dixon sequences are more appropriate for assessing whether treatment has led to an actual change in fat content. The T2w-TSE Dixon warrants further validation for the source of its sensitivity to lesions. In the absence of lesion relaxation time characterisation, the many confounders affecting this technique make it difficult to draw conclusions regarding its potential benefits. With regards to SAR mitigation strategies for a TSE-based method employed at high field (i.e., > 1.5 T), it is advised to keep the repetition time the same by instead altering the refocusing angle. This will have a marginal effect on fat fraction estimation and overall image contrast. In contrast, modification of echo train length and number of slices may have slice profile or magnetisation transfer effects. The latter can result in the water signal being suppressed, resulting in a higher estimated fat fraction [37].

The limitations of this study include the small number of lesions included in the analysis, which limits the exploratory assessment of the quantitative measures, particularly T1 and R2*, and should be investigated further in a larger study. T1_water_ was not reconstructed with a B_1_ correction, and the flip angles chosen may not be ideal for the T1 range of both active and treated bony lesions [38], which may confound estimates of T1_water_. Furthermore, the spectral model used in the q-Dixon fat fraction estimates was optimised for liver and the spectral model of normal-appearing marrow for patients with advanced prostate cancer remains unknown. The q-Dixon estimates may, therefore, not be an optimal reference in this study. Despite this, the spectrum of healthy bone marrow fat has been shown to be similar to that of liver fat [39], and a previous study in liver has demonstrated that liver fat quantification is relatively insensitive to the choice of spectral model provided the model chosen considers multiple fat resonances [40]. Whilst not investigated in this study, the TSE sequences used in this study will be subject to signal modulations due to the J-coupling of fat [41]. The results presented in this study were acquired at a single field strength (1.5 T). Additional challenges at higher field may make the results in this study fail to generalise. These include the increase of T1 with field strength, resulting in altered T1-related bias. Accelerated T2* dephasing at 3 T will also introduce its own relaxation-related bias and noise sensitivity. Finally, the need for tighter echo spacings may require interleaved acquisitions for gradient echo-based methods due to the accelerated phase cycling of fat and water, which will depend on the specification of available gradient hardware.

In conclusion, the results of this study indicate that PDw methods offer different results compared to T1w- and T2w-TSE Dixon sequences when interrogating the tissue fat concentration of tissues within the pelvis, including bony lesions. If considering a choice of Dixon sequence for fat quantification in whole-body imaging, it is most reasonable to select a gradient echo approach owing to their short acquisition time being amenable for a single-breath hold. For minimising sources of bias, PDw approaches are preferred, particularly options that offer multispectral fat modelling and T2* correction. Whether these methods have the same sensitivity to treatment effects as a T1w-Dixon remains undetermined. Future work using spectroscopy to characterise the relaxation times, alongside the fat content of involved bone marrow, could provide necessary clarity and motivation for improved quantitative imaging methods in oncology for bone marrow disease.

## Supplementary information

ELECTRONIC SUPPLEMENTARY MATERIAL

## Abbreviations

ADC: Apparent diffusion coefficient
DWI: Diffusion-weighted imaging
FF: Fat fraction
MET-RADS-P: METastasis Reporting and Data System for Prostate Cancer
PD: Proton-density
PDFF: Proton density fat fraction
PDw: Proton-density weighted
ROI: Region of interest
SAR: Specific absorption rate
T1w: T1-weighted
T2w: T2-weighted
TSE: Turbo spin echo
WB-MRI: Whole-body MRI
