# Supplementary material for: Influence of imaging method on fat fraction estimation for assessing bone marrow in metastatic prostate cancer
Source: Eur Radiol. 2025 Apr 11;35(10):6039–51. doi: 10.1007/s00330-025-11564-7 (PMC12417236; doi:10.1007/s00330-025-11564-7)
Supplement: Supplementary file 1 — ELECTRONIC SUPPLEMENTARY MATERIAL [file 330_2025_11564_MOESM1_ESM.pdf]

Influence of imaging method on fat fraction estimation for assessing bone marrow in metastatic prostate cancer

ELECTRONIC SUPPLEMENTARY MATERIAL

An initial phantom experiment was performed to determine the feasibility of utilising 2D turbo spin-echo (TSE) Dixon sequences with contiguous slices whilst achieving minimal cross-talk. The phantom was constructed using peanut oil, based on the recipe reported by Zhao et al [1] with vials containing fat fractions from 0 to 100%. Fat fraction estimations were compared between a 3-point T2-TSE Dixon without a slice gap and two interleaved T2-TSE Dixon acquisitions with a slice gap equal to the slice thickness.

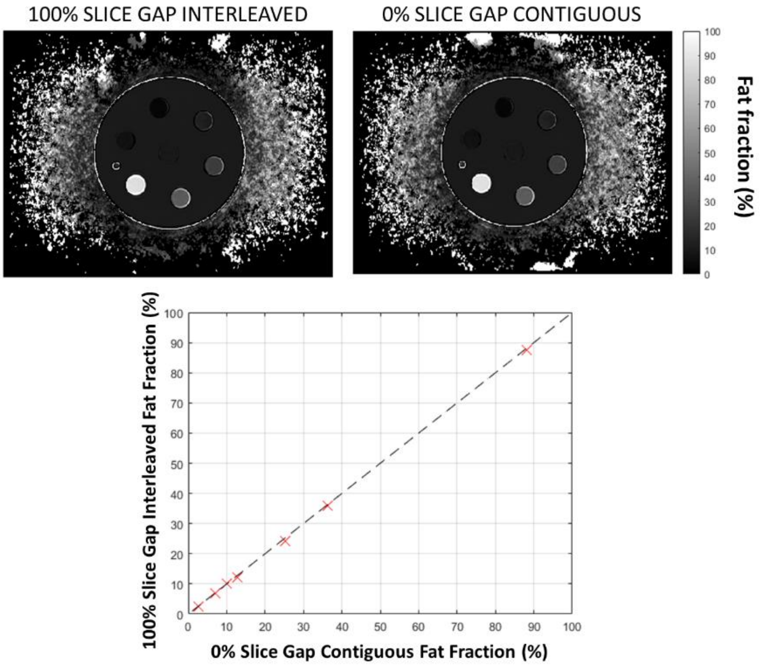

Figure 1: Fat fraction maps acquired with different methods of achieving contiguous slices. From left to right starting from the top left corner of both fat fraction maps, the seven fat vials within the phantom contain nominal fat fraction values of 10%, 20%, 5%, 0%, 30%, 100%, 40%. Depicted in the plot below is the measured fat fraction with two interleaved sequences each with a slice gap equal to the slice thickness and offset and a single sequence without a slice gap.

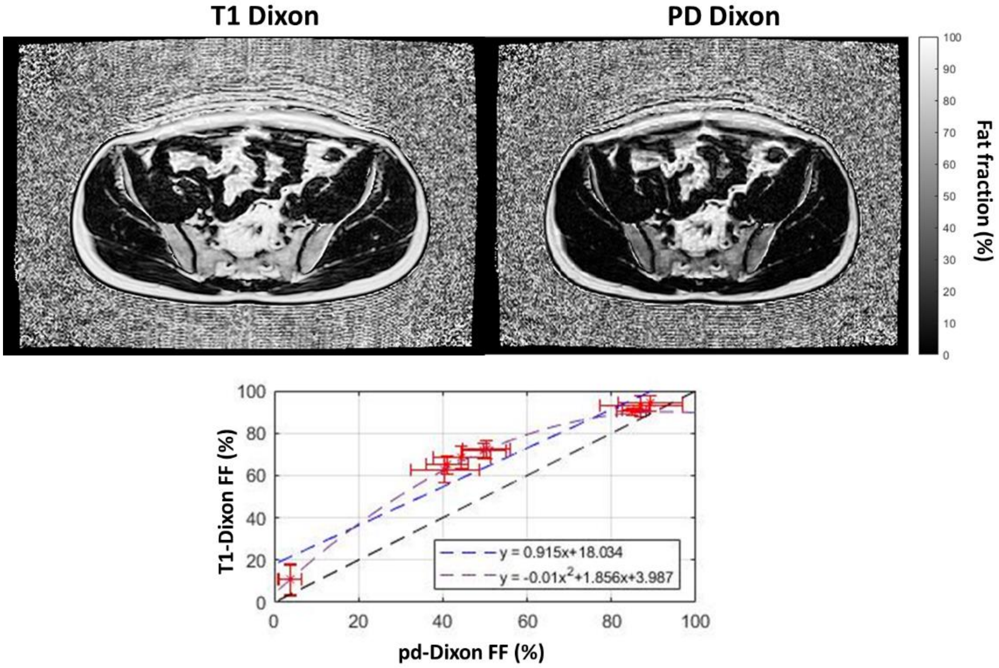

Figure 2: Fat fraction maps acquired with different flip angles in 2-point gradient echo Dixon sequences to achieve different contrast weightings. ROIs were drawn in the left and right gluteal muscle, posterior iliac bones, subcutaneous fat, and femoral heads. Depicted in the plot below is the agreement between the fat fraction values of both contrasts.

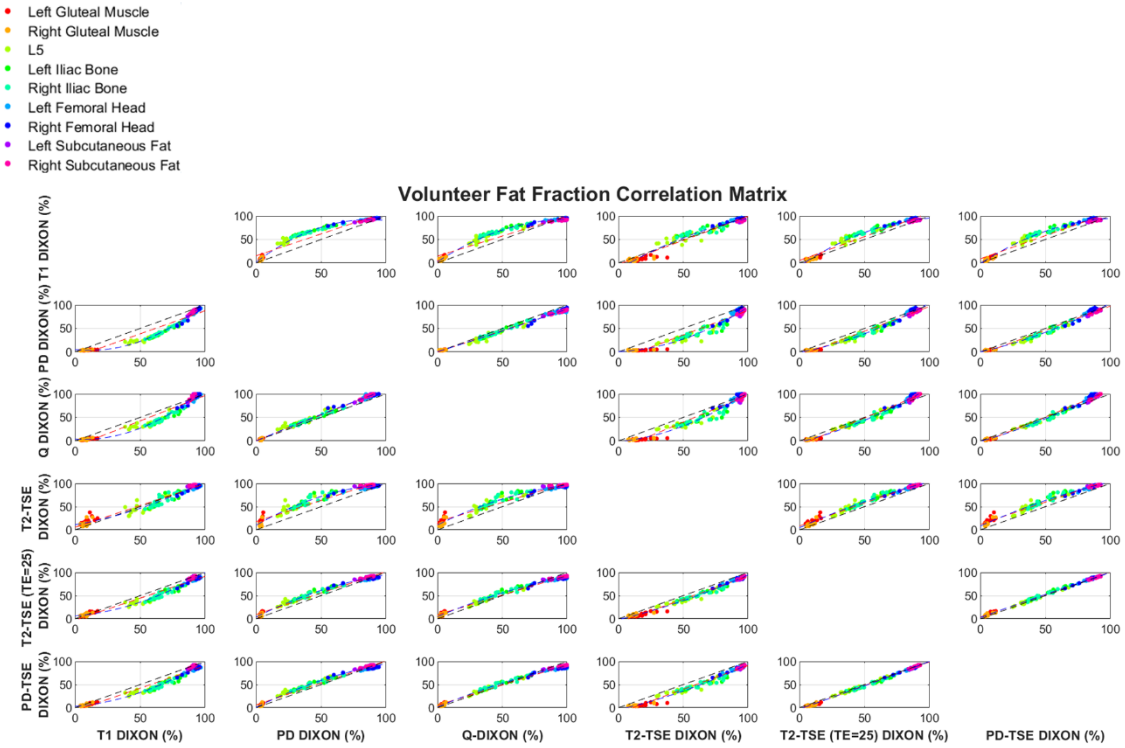

Figure 3: Complete volunteer fat fraction correlation matrix.

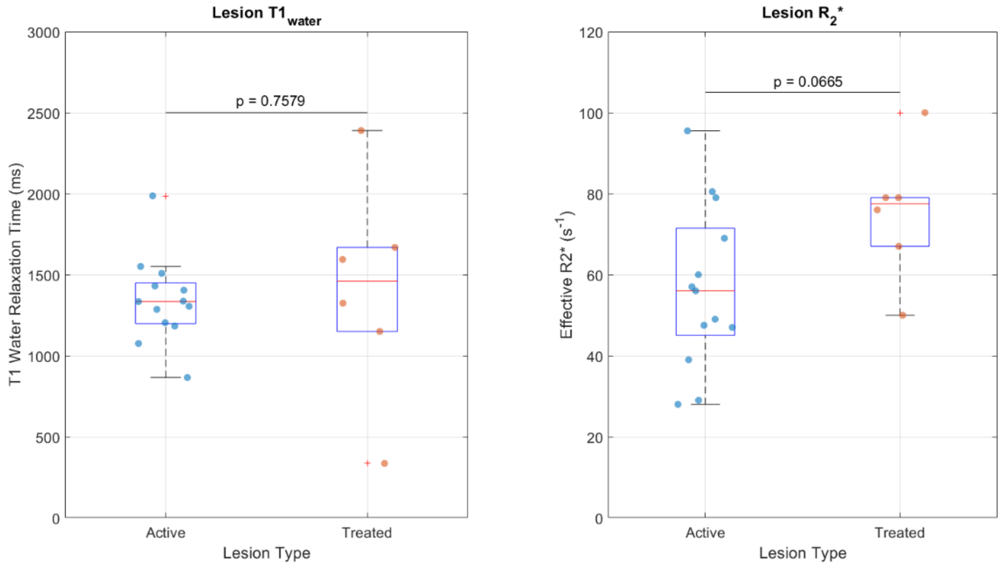

Figure 4: Relaxometry results for active and treated lesions

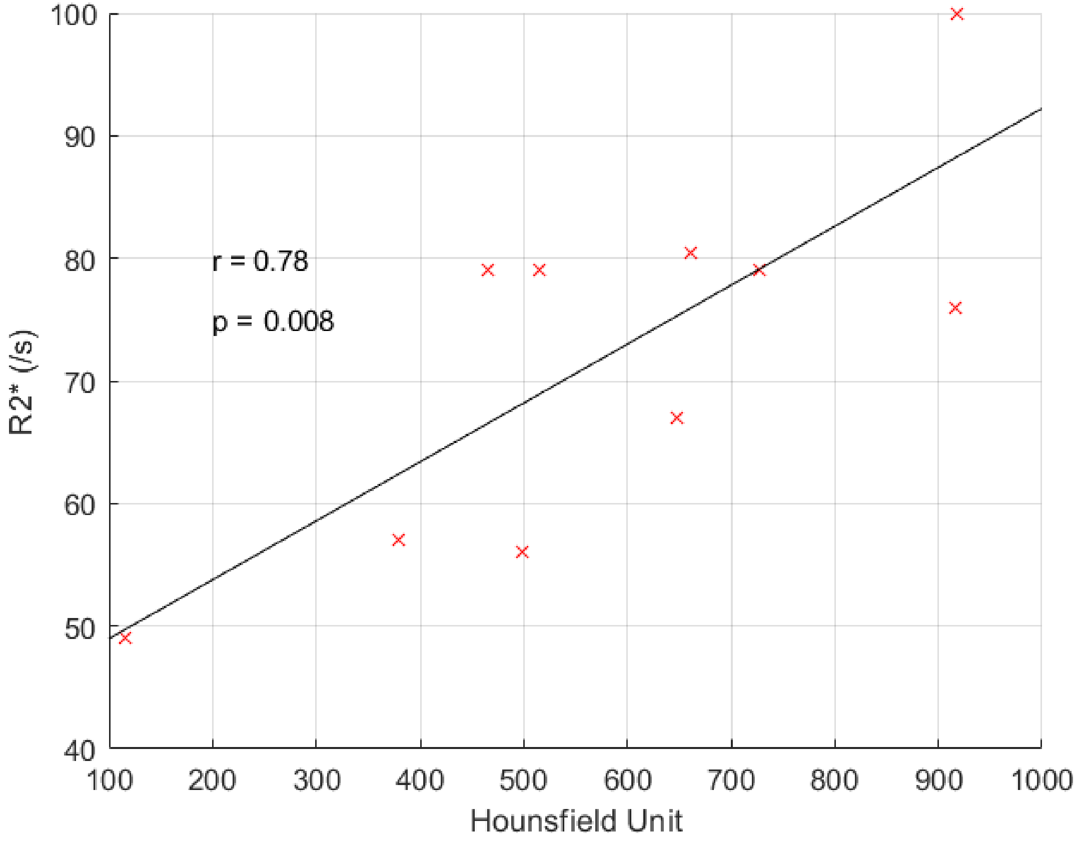

Figure 5: Correlation between R2* and Hounsfield Unit for lesions where CT and MRI were acquired within three months of each other.

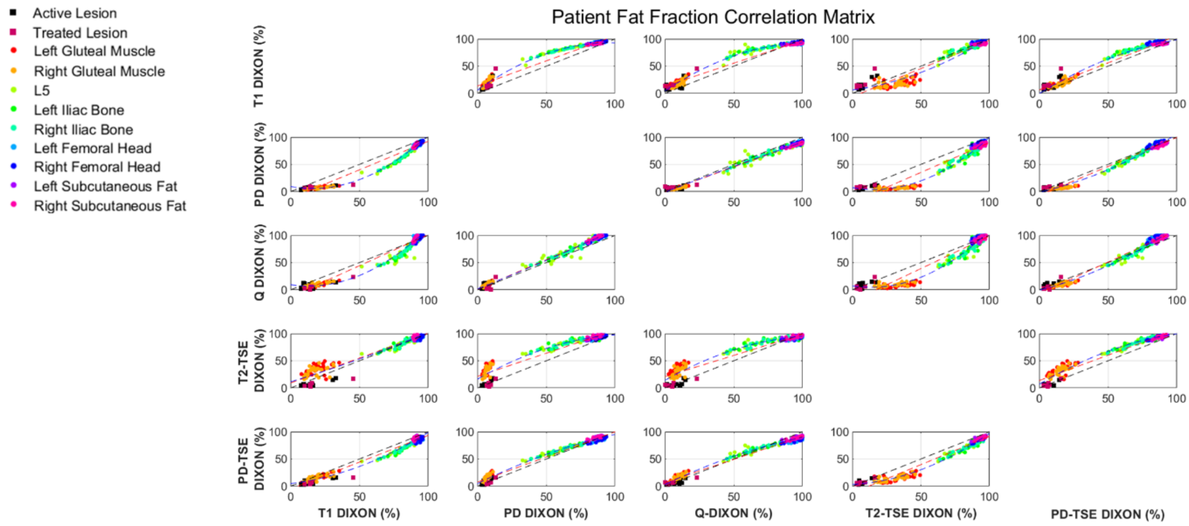

Figure 6: Complete patient fat fraction correlation matrix.

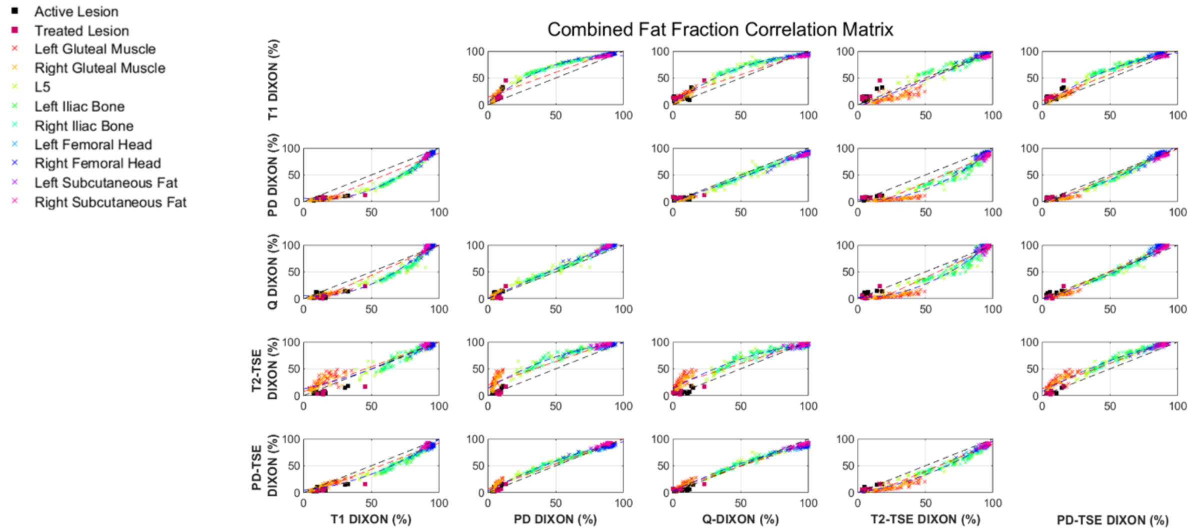

Figure 7: Complete volunteer and patient pooled fat fraction correlation matrix.

1 Zhao R, Hamilton G, Brittain JH, Reeder SB, Hernando D (2021) Design and evaluation of quantitative MRI phantoms to mimic the simultaneous presence of fat, iron, and fibrosis in the liver. Magnetic Resonance in Medicine 85:734-747
